# Understanding the motivations of patients: A co‐designed project to understand the factors behind patient engagement

**DOI:** 10.1111/hex.12942

**Published:** 2019-08-04

**Authors:** Tamara L. McCarron, Thomas Noseworthy, Karen Moffat, Gloria Wilkinson, Sandra Zelinsky, Deborah White, Derek Hassay, Diane L. Lorenzetti, Nancy J. Marlett

**Affiliations:** ^1^ The Department Community Health Sciences Calgary Alberta Canada; ^2^ O’Brien Institute for Public Health Calgary Alberta Canada; ^3^ University of Calgary Calgary Alberta Canada; ^4^ Faculty of Nursing University of Calgary Doha Qatar; ^5^ Haskayne School of Business Calgary Alberta Canada; ^6^ Health Sciences Library University of Calgary Calgary Alberta Canada

**Keywords:** co‐design, health care reform, motivation, patient participation

## Abstract

**Background:**

Large‐scale transformation depends on effective engagement of diverse stakeholders. With the evolution of the role of the ‘patient partner’ in health‐care decision making, understanding the motivations of these individuals is essential to the success of engagement initiatives. This study reports on motivational factors associated with patient engagement in health care.

**Methods:**

Patient co‐investigators and a researcher co‐designed and conducted this study. A survey was administered to patients and family members. Key informant interviews and previous research informed the development of the survey tool. The survey data were analysed using exploratory factor analysis to identify the underlying dimensions in the data. Cronbach's alpha was used to determine reliability.

**Results:**

A total of 1449 individuals participated in the survey. Of these, 543 completed and 427 partially completed the survey (67% complete rate). The mean age of the respondents was 54 years. The majority of participants were female, well‐educated, retired, married and lived in an urban centre. Seven motivational factors explained 65% of the total variance. Analysis of internal consistency revealed acceptable reliability for all items. The seven motivations were as follows: *Self‐fulfillment*, *Improving Healthcare*, *Compensation*, *Influence*, *Learning New Things*, *Conditional and Perks.*

**Conclusion:**

The results of this research describe a sample of patient and family members currently engaged with health systems. We identified seven motivational factors underlying their engagement. A deeper knowledge of volunteer motivations will not only create meaningful engagement opportunities for patients, but also enable health organizations to gain from the experience of these individuals, thereby enhancing quality and sustainability of patient engagement programmes.

## 1 BACKGROUND

Large‐scale health‐care transformation requires the effective engagement of all stakeholders across the entire health system, including providers and patients.^1^, ^2^ Even though patient engagement has gained attention as an approach to improving the quality, safety and service delivery of health care, uncertainty still persists about if, why and how to involve patients in effective and meaningful ways.^3^, ^4^ Coupled with a lack of evidence, initiatives to engage patients in health‐care decision making are infrequent and have also demonstrated limited success.^5^, ^6^, ^7^, ^8^ Accordingly, more knowledge is needed with respect to effective approaches to attracting and sustaining patients' participation in health‐care decision making.^9^, ^10^, ^11^, ^12^ For the purpose of this manuscript, a stakeholder is defined by persons involved with or affected by a course of action.^13^ In this manuscript, we focus on the central stakeholder in health care, patients and family members.

Motivation is defined as the force that occurs when a need is aroused that an individual is driven to satisfy.^14^, ^15^, ^16^, ^17^, ^18^ The measurement and exploration of volunteer motivation is not new to researchers.^19^, ^20^, ^21^, ^22^, ^23^, ^24^, ^25^, ^26^ Many scholars have applied a variety of theories to attempt to understand why people volunteer.^27^, ^28^, ^29^, ^30^, ^31^, ^32^, ^33^ A deeper understanding of these motivations, specific to health care, will not only advance the development of effective strategies for patient engagement but also inform approaches to measuring the impact and overall effectiveness of these initiatives. Recognizing that people often pursue multiple, competing goals, they not only strive to achieve the desired outcomes but also attempt to maximize the value they receive.^34^ Since individuals have limited time and resources, they often have to choose what choice to prioritize. Simply speaking, individuals initially form a ‘consideration set’ consisting of ‘alternatives from which choice is made’,^35^, p. 522. These choices are complex and unique to the individual making them.^35^, ^36^, ^37^ The market choice behaviour (MCB) theory is the amalgamation of conceptual frameworks drawn from economics, psychology, sociology, marketing and consumer behaviour. Sheth et al postulate that this theory explains consumer choice behaviour.^14^, ^15^, ^27^, ^28^, ^36^, ^38^, ^39^ MCB is universal and central to this decision making is the allocation of three precious resources: money, time and effort. Since we are interested in understanding the motivations of people who engage with health‐care organizations to inform a patient engagement framework, we can use this theory to understand how individuals make time allocation choices.^36^

The Patient and Family Advisory Group (PFG) in Alberta, Canada, was formed in 2010 with the purpose of enhancing the patient experience by improving the quality and safety of patient care. Since this time, opportunities for individuals to assume roles as ‘patient advisors’ have evolved and are continuing to evolve, not only within Alberta Health Services, but also in other health organizations such as the Health Quality Council of Alberta.^40^ These individuals, serving in various roles, engage in decision making opportunities, in not only service delivery but also other health‐care transformation activities, including governance. These individuals primarily ‘volunteer’ their time. However, some individuals receive stipends, honoraria and expense reimbursements. Understanding how and what motivates these individuals is currently unknown. Using MCB as a theoretical framework to guide the development of the survey, this study will understand the motivations of individuals engaging with health organizations in various roles such as patient advisors. This paper reports on the findings of a provincial survey to understand patient motivations and, second, provides the foundation for the development of a framework for patient motivation.

## 2 METHODOLOGY

### 2.1 Co‐design and patient co‐investigators

This study utilized a co‐design methodology, wherein individuals who share an interest in the outcome of this research, in this case the patient and family community, were involved in the design and delivery of the research, from project inception to final analysis and write‐up.^41^, ^42^ These patients were selected based on their prior experiences and ability to commit to the project. Three patient co‐investigators and the first author formed the project team.

### 2.2 Sampling strategy

We began with the assumption that ‘patient partners’ exist in many organizations, and can assume a myriad of roles including advocate, advisor and volunteer. To ensure a diverse sampling strategy, and facilitate provincial‐wide participation, we compiled a comprehensive list of all organizations in Alberta, Canada, known to have patient engagement programmes. We then snowball sampled from among key informants, with organizations identifying other individuals, such as Alberta Children's Hospital and the Health Quality Council of Alberta (see Table 1). These key informants were asked to assist in recruiting participants within their respective organizations. Participants were included if they self‐identified as having experiences participating in engagement programmes (eg, as a patient advisor with Alberta Health Services), were fluent in English, lived in Alberta and were over 18 years of age.

**Table 1:** Participating organizations

Organizations	Location
Alberta Rural Development Network	Edmonton
Alberta Stollery Children's Hospital	Edmonton
Cancer Control Board	Calgary
Patient and Family Advisory Committee	Edmonton
Glenrose Rehabilitation Hospital	Edmonton
Alberta Health Services—Provincial Volunteer Resources	Calgary
Alberta Children's Hospital	Calgary
South Health Campus	Calgary
Patients 4 Change	Calgary
Covenant Health	Edmonton
Alberta SPOR Network	Edmonton
Patient and Community Engagement Researcher (PaCER)	Calgary
Health Quality Council of Alberta	Calgary
Wellspring	Calgary
Alberta Health Services—Strategic Clinical Networks	Calgary
Alberta Health Services—Primary Care Network	Calgary
Imagine Citizens	Calgary
Choosing Wisely Alberta	Calgary
Open Arms Advocacy	Calgary
Alberta Arthritis Association	Calgary
Alberta Health Services—South Zone	Lethbridge
Alberta Health Services—North Zone	Grande Prairie

### 2.3 Ethics approval

This study was approved by the Conjoint Health Research Ethics Board (CHREB) at the University of Calgary. Possible participants were given background information about the project and the purpose of the study. Individuals were also encouraged to contact the researchers if they had any questions prior to participating.

### 2.4 Patient and family interviews

To support the development of the survey tool, individuals with prior experience partnering with one of the organizations of interest were recruited, using purposive sampling, and were selected based on their experiences as a patient or family member. An interview questionnaire was developed to understand the underlying motivations of individuals in deciding to get involved, the perceived impact they had, contributions they felt they had made, and what they thought was needed to support their continued involvement. Twenty‐three semi‐structured interviews, with an open‐ended question style, lasting an average of 1 hour, were conducted between May and December 2017. Each of the interviews was analysed using a modified constant comparative method developed by Glaser.^43^ This required a side‐by‐side comparison and analysis of the transcripts from each of the interviews to determine common themes.

### 2.5 Survey development

The survey was developed in collaboration with the research team and structured to facilitate a broader understanding of the motivations of patients and family members.^44^ The interviews, previous studies focusing on volunteer motivation and the results of a scoping review 45 informed the survey tool. A model developed by Sheth et al^36^ provided a deeper understanding of five consumer motivations for choice behaviour. This understanding assisted the research team in selecting response statements to inform the survey questions.

### 2.6 Survey pilot testing

Prior to launch, a convenience sample of five patient and family members tested the survey tool. Each reviewer was asked to assess the relevance and quality of each item, and to identify unnecessary or ambiguous questions (including clarity, relevance, flow and wording).^44^ The survey took approximately 20 minutes to complete.

### 2.7 Survey instrument

The final survey consisted of 30 questions, organized into eight sections, with response options that included a combination of open‐ended text, multiple choice and a series of a 5‐level Likert‐scale questions. The survey can be found in Supporting Information.

### 2.8 Survey administration

The survey was administered online, using a tool called REDCap,^46^ between 7 March and 27 April 2018. Key informants were contacted by phone and, later, via email to notify them of the survey launch. The invitation email included a message which informed possible participants of the purpose of the study and included a link to the online survey. In order to participate, individuals had to answer yes to three questions: (a) Are you a patient (chronic or occasional user of the health system) or family member? (b) Are you currently (or have you previously been) a volunteer with health organizations in various roles including (but not limited to) an advisor, a researcher, a navigator or a committee member? and (c) Are you able to participate in an online survey that would take approximately 20 minutes to complete? Information on the study was distributed by key informants to possible participants using email, electronic newsletters or through the organization's websites or social media accounts. No patient contact information was shared with the research team. A poster was also developed with similar information as the email so that this opportunity could be displayed on notice boards when requested. Paper copies of the survey were made available upon request, and mailed, with a self‐addressed stamped envelope, to individuals wishing to participate offline. The survey was posted for 7 weeks. On three occasions, participating organizations were asked via email to remind potential participants of the study: after the second week, the final week and the last day.

### 2.9 Data analysis

Survey data were analysed using a statistical analysis program (SPSS 25). The demographic information (section 7) and survey responses (Q2.1, 2.2, 2.3, 2.4, 2.5, 2.7, 2.8, 3.1, 4.1, 4.2 and 6.3) were analysed using descriptive statistics. Likert‐scale questions (Q3.2, 3.3, 3.4, 4.3, 5.1, 5.2, 6.1, 6.2, 6.5), consisting of 62 statements, were analysed using exploratory factor analysis. To reduce measurement error, reverse coding was used.^47^, ^48^ We chose to use principal axis factoring as our extraction method in an attempt to identify the underlying dimensions of the data.^49^, ^50^, ^51^ In order to determine whether we should conduct factor analysis, four key pieces of information were considered: the sample size^50^, ^52^, ^53^, ^54^; the correlation matrix^53^, ^54^, ^55^; the Kaiser‐Meyer‐Olkin test of sampling adequacy,^56^, ^57^, p. 112; and Barlett's test of sphericity.^58^ Since the purpose of our research was to identify items that were strong indicators of patient motivation, we decided to remove communalities with magnitudes <0.4^50^, ^55^; factor loadings with <2 variables^50^, ^55^; and variables that cross‐loaded.^50^, ^55^ Although the minimum acceptable standard for factor loadings is typically 0.32, to improve factor interpretation we elected to define a cut‐off >0.50.^55^, ^59^ Guttman‐Kaiser's criterion, a Scree test and the proportion of variance assisted in determining the number of factors to retain.^60^, ^61^, ^62^, ^63^ Varimax and direct oblimin produced similar solutions, so we reported data from the varimax rotation.^49^, ^50^, ^52^, ^53^, ^55^ The internal consistency of the scale items was established using Cronbach's alpha (*α*).^52^, ^64^

## 3 RESULTS

### 3.1 Patient and family interviews

Analysis of the patient and family interviews revealed three distinct themes that describe a path for patient and family engagement. These three themes, the recruit theme (why participants got involved), the retain theme (why participants continue to be involved) and the sustain theme (what participants need to strengthen their involvement), informed the structure of the survey, and questions were developed to better understand the motivations at each of these themes (see Supporting Information for the survey).

### 3.2 Characteristics of survey respondents

A total of 1449 individuals participated in the survey. Of these, 543 completed and 427 partially completed the survey (67% complete rate). All returned surveys, including partially completed surveys, were analysed. In order to accurately capture the motivations of individuals currently engaged as ‘patient partners’, participants who indicated they were ‘no longer involved’ or ‘currently taking a break’ (question 2.2) were removed from further analysis (n = 114). The remaining respondents self‐identified as actively involved in their roles (n = 856). These active participants had an average age of 54 years, with the youngest being 18 and oldest being 91 years of age. These individuals were primarily female (n = 393, 80%), retired (n = 208, 34%), married (n = 288, 59%) and living in an urban centre (n = 299, 63%). Respondents were well‐educated with 29% (n = 143) having an undergraduate degree, 29% were college‐educated (n = 142) and 18% had a graduate degree (n = 89). Thirty‐three percent of respondents (n = 164) indicated they were former health‐care employees (see Table 2).

**Table 2:** Participant characteristics

(Frequency, %)	
Gender (n = 489)	
Male	92 (19)
Female	393 (80)
Prefer not to answer	3 (0.6)
Other	1 (0.4)
Age (n = 473)	
Under 20	30 (6)
21‐35	74 (16)
36‐50	65 (14)
51‐65	152 (32)
66‐80	136 (29)
81+	16 (3)
Highest level of education (n = 490)	
Primary/Elementary	1 (1)
High school	85 (17)
College	142 (29)
University—Bachelor	143 (29)
University—Graduate	89 (18)
None	0 (0)
Prefer not to answer	9 (2)
Other	21 (4)
Marital status (n = 490)	
Single	87 (18)
Married (and not separated)	288 (59)
Common law	21 (4)
Separated, but still legally married	6 (1)
Divorced	25 (5)
Widowed	47 (10)
I prefer not to answer	12 (2)
Other	4 (1)
Employment status (n = 613)	
Full time	106 (17)
Part time	75 (12)
Caregiver	37 (6)
Homemaker	44 (7)
Student (full time)	54 (9)
Student (part time)	16 (3)
Self‐employed	29 (5)
Receiving disability benefits	21 (3)
Retired	208 (34)
Prefer not to answer	4 (1)
Other	19 (3)
Where do you live? (n = 477)	
Urban	299 (63)
Rural	178 (37)
What role best describes you? (n = 851)	
Volunteer	539 (63)
Advisor	99 (12)
Advocate	42 (5)
Researcher	11 (1)
Patient and Community Engagement Researcher (PaCER)	39 (5)
Other	121 (14)
How did you hear about this role? (n = 739)	
I received an email	97 (13)
I was personally asked by my physician or health‐care provider	39 (5)
I was contacted directly by an employee with (Alberta Health Services, Alberta Children's Hospital, etc)	85 (12)
My friend or family member told me about it	131 (18)
I saw a poster or advertisement	71 (10)
I searched out the opportunity myself	240 (32)
Other	76 (10)
How did you learn about what was required of you in this role? (n = 1135)	
I participated in training	282 (25)
I went through a selection process (an interview and/or application form)	285 (25)
I attended an information/orientation session	250 (22)
I researched the opportunity online	91 (8)
My friend told me about it	84 (7)
I was asked by my physician to consider the opportunity	29 (3)
Other	114 (10)
Current or previous health‐care employee (n = 488)	
Yes	164 (33)

### 3.3 Active participant roles

When asked what role best described them, 63% of respondents (n = 539) identified themselves as a ‘volunteer’. When asked how they heard about their role, 32% of respondents (n = 240) indicated they searched out the opportunity themselves and 18% (n = 131) indicated a friend or family member told them about it. When asked how they learned about what was required of them for the role, 25% (n = 282) of respondents participated in training and an additional 25% (n = 285) indicated they participated in a selection process. When asked, on average, how much time they spend in their role, respondents indicated they volunteered an average of 16 hours/wk, with the average tenure in their various roles of 4 years. Seventy‐seven percentage (n = 461) of respondents indicated that, if asked, they would be willing to give more time to their roles. When asked whether they would like to continue in their current roles, 98% of respondents (n = 489) answered ‘yes’ (see Table 2).

### 3.4 Participant experience

When asked how they felt about their role, 53% (n = 324) of respondents strongly agreed that they feel ‘interested’, in their role as a patient partner. Fifty percent (n = 316) strongly agree they experience a sense of pride in their role. Forty‐seven percent of respondents (n = 294) strongly agreed they feel ‘happy’, 42% of respondents (n = 262) feel ‘stimulated’, and 9% feel ‘underutilized’ (n = 54) in their role. Respondents were asked about their overall experience while serving in their respective roles. When asked, ‘In this role, I feel ________’, respondents strongly agreed that they feel ‘appreciated’ (n = 204, 38%), ‘have made a difference’ (n = 179, 33%), ‘that they feel valued’ (n = 169, 32%), ‘needed’ (n = 169, 32%), ‘engaged’ (n = 163, 31%), ‘excited’ (n = 148, 28%), ‘included’ (n = 140, 26%), ‘challenged’ (n = 128, 24%) and ‘important’ (n = 118, 22%). A small percentage of respondents also ‘strongly agreed’ they felt ‘unappreciated’ (n = 8, 2%) and they had ‘wasted their time’ (n = 7, 1%). When asked whether ‘doing this work gives them a sense of ________’, respondents strongly agreed that the work gave them a sense of ‘purpose’ (n = 214, 40%), ‘inspiration’ (n = 174, 33%), ‘hope’ (n = 162, 31%), ‘connection’ (n = 146, 28%), ‘knowledge’ (n = 138, 26%), ‘competence’ (n = 112, 21%) and ‘empowerment’ (n = 90, 17%). A small percentage of respondents also ‘strongly agreed’ that the role gave them a sense of ‘frustration’ (n = 12, 2%) and ‘aggravation’ (n = 8, 1%; see Table 3).

**Table 3:** Frequency table for participant experience in role

	Strongly disagree (frequency, %)	Disagree (frequency, %)	Neither agree nor disagree (frequency, %)	Agree (frequency, %)	Strongly agree (frequency, %)
How do you feel about your role?					
I feel proud	7 (1)	3 (0)	85 (14)	218 (35)	316 (50)
I feel happy	6 (1)	6 (1)	66 (11)	250 (40)	294 (47)
I feel stimulated	6 (1)	17 (3)	83 (13)	254 (41)	262 (42)
I feel interested	5 (0)	7 (1)	23 (4)	258 (42)	324 (53)
I feel underutilized	110 (18)	177 (29)	163 (27)	101 (17)	54 (9)
In this role, I feel____________					
Needed	8 (1)	16 (3)	63 (12)	279 (52)	169 (32)
I have wasted my time	309 (58)	155 (29)	46 (9)	14 (3)	7 (1)
Appreciated	5 (1)	8 (1)	47 (9)	273 (51)	204 (38)
Excited	5 (1)	19 (4)	131 (24)	232 (43)	148 (28)
Challenged	15 (3)	44 (8)	126 (24)	217 (41)	128 (24)
Important	6 (1)	27 (5)	177 (33)	205 (39)	118 (22)
I have made a difference	4 (1)	10 (2)	75 (14)	268 (50)	179 (33)
Valued	5 (1)	13 (2)	55 (10)	290 (55)	169 (32)
Unappreciated	281 (53)	151 (28)	72 (13)	22 (4)	8 (2)
Included	6 (1)	20 (4)	109 (20)	263 (49)	140 (26)
Engaged	5 (1)	16 (3)	73 (14)	271 (51)	163 (31)
Doing this work gives me a sense of___________?					
Purpose	3 (1)	9 (2)	37 (7)	270 (50)	214 (40)
Aggravation	250 (48)	156 (30)	66 (13)	40 (8)	8 (1)
Competence	4 (1)	19 (4)	111 (21)	280 (53)	112 (21)
Knowledge	4 (1)	19 (3)	59 (11)	311 (59)	138 (26)
Empowerment	23 (4)	44 (9)	175 (33)	194 (37)	90 (17)
Inspiration	5 (1)	17 (3)	77 (15)	253 (48)	174 (33)
Frustration	217 (42)	147 (28)	85 (16)	63 (12)	12 (2)
Connection	4 (1)	13 (2)	64 (12)	303 (57)	146 (28)
Hope	7 (1)	14 (3)	93 (18)	248 (47)	162 (31)

### 3.5 Patient motivations

The factor loadings and the corresponding variables within their factor loadings can be found in Table 4. Seventeen variables were removed from our analysis: ten as a result of low communalities; four to poor factor loadings; and three to cross‐loadings. Seven of the eigenvalues were over Guttman‐Kaiser's criterion of 1. The scree plot indicated a clear break after the seventh factor.^60^, ^61^, ^63^, ^65^, ^66^ The seven identified motivations that explain 65% of the total variance in engagement were named: *Self‐fulfillment* 27.2%, *Improving Healthcare* 13.8%, *Compensation* 7.7%, *Influence* 5.4%, *Learning New Things* 4.5%, *Conditional* 3.4% and Perks 3.0%. Analysis of internal consistency using Cronbach's alpha revealed acceptable reliability for all seven motivations: *Self‐fulfillment* (0.901), *Improving Health‐care* (0.886), *Compensation* (0.894), *Influence* (0.871), *Learning new things* (0.894), *Conditional* (0.809) and *Perks* (0.826) (see Table 5).

**Table 4:** Active participant factor loadings after varimax rotation (n = 856)

Variables	Items	Components						
		Factor 1	Factor 2	Factor 3	Factor 4	Factor 5	Factor 6	Factor 7
V99	I am making a difference	0.806						
V114	A sense of purpose	0.745						
V76	I enjoy what I am doing	0.717						
V77	Helping others	0.71						
V91	I feel I am making a difference	0.685						
V107	I see the difference I am making	0.644						
V96	I am supporting other patients	0.621						
V95	I have established important relationships	0.611						
V101	I have improved patient experience	0.581						
V48	To make health‐care better		0.816					
V43	Improving the health‐care system		0.814					
V39	I want to improve the health‐care system		0.765					
V49	To change the current culture of health care		0.667					
V79	Helping to improve health care		0.616					
V36	I want to improve health care for myself and my family		0.565					
V51	To speak for those who cannot speak for themselves		0.531					
V103	I am getting paid			0.876				
V42	Earning extra money			0.844				
V80	I get paid			0.843				
V34	It is an opportunity to make some extra money			0.81				
V110	I receive payment			0.649				
V35	I get to travel			0.583				
V89	I am challenging the ‘norm’				0.712			
V88	I am impacting decisions				0.697			
V90	I am paving the way for others				0.619			
V86	Others listen to me				0.538			
V93	Communication between patients/family members and health professionals has improved				0.511			
V38	I get to learn new things					0.776		
V44	Learning new things					0.751		
V81	I learn new things					0.723		
V115	I continue to learn new things					0.642		
V124	Your expenses are reimbursed						0.738	
V125	You can work from home						0.71	
V128	You attend an annual conference						0.686	
V127	The role could turn into a paid position						0.622	
V122	The commitment requires that you only attend four meetings per year						0.591	
V116	My expenses are paid							0.862
V84	My expenses are paid							0.73
V111	I am able to travel							0.496

Note

Extraction method: principal axis factoring.

Rotation: varimax with Kaiser normalization.

Rotation converged in 8 iterations.

Factor loadings under 0.50 suppressed.

**Table 5:** Summary statistics for patient motivations

Motivation	Eignevalues (%)	Number of items	Cronbach's alpha	Mean	Variance	Standard deviation
Self‐fulfillment	27.2	9	0.901	36.84	28.91	5.4
Improving Healthcare	13.8	7	0.886	27.6	32.5	5.7
Compensation	7.7	6	0.894	8.43	15.15	3.89
Influence	5.4	5	0.871	17.12	15.03	3.88
Learning new things	4.5	4	0.894	16.5	9.15	3.02
Conditional	3.4	5	0.809	16.26	22.62	4.76
Perks	3	3	0.826	5.82	10	3.16

### 3.6 Self‐fulfillment

The self‐fulfillment motivation includes nine variables. These variables can be organized into four categories: helping others (v77, v96 and v101), the overall gratification received from the opportunity (v76, v91, v99 and v107), meaningful connections (v95) and a sense of purpose (v114).

### 3.7 Improving healthcare

The improving healthcare motivation includes seven variables. These motivations are defined by the desire to improve the health‐care system (v36, v39, v43, v48, v79), to improve the current culture of health care (v49) and to speak for those who are not able to speak for themselves (v51).

### 3.8 Compensation

The compensation motivation is unique as it is an example of individuals being motivated financially. Monetary in nature, this motivation can be in the form of a stipend or honorarium (v34, v35, v42, v80, v103 and v110). This motivation includes six variables.

### 3.9 Influence

The influence motivation defines the ability of an individual to affect change and to have a perceived impact on the health system, or the health‐care professional or decision maker with whom they work. This motivation includes five variables which describe being listened to (v86), the ability to impact decisions (v88 and v93) and the ability to be a proponent for change (v89 and v90).

### 3.10 Learning new things

The learning new things motivation describes an individual's desire to learn and continue to learn new things. It includes four variables (v38, v44, v81 and v115).

### 3.11 Conditional

The conditional motivation is contingent on the specific situation faced by the individual. These motivations often enhance the choice of the individual, to engage or not to engage, and are usually situationally dependent. For example, someone who lives remotely could decline to participate in a face‐to‐face meeting due to the need to travel. This motivation has five variables and is described by the need for flexibility such as ‘you can work from home’ (v122 and v125) and the potential scenarios for the role such as ‘the role could turn into a paid position’ (v124, v127 and v128).

### 3.12 Perks

The perks motivation has three variables and is another example of being motivated by extra benefits beyond being financially compensated. Perks include things such as having expenses paid (v84 and v116) or being supported to attend conferences (v111).

These seven factors represent the underlying motivations of engagement for a sample of patient and family members currently engaged with health systems in Alberta.

## 4 DISCUSSION

We undertook this study to better understand the motivations of individuals who choose to give their time and talents to health organizations. The results of our provincial survey depict these individuals as primarily well‐educated, female, retired and living in an urban location. The majority of respondents described themselves as volunteers who sought out the opportunity themselves. Respondents were generally pleased with their roles, indicating they felt a sense of pride being in these roles and they felt that these opportunities provided a sense of purpose. We used the results of this survey to explore motivations for patient engagement. The motivations we identified were as follows: *Self‐fulfillment*, *Improving Healthcare*, *Compensation*, *Influence*, *Learning New Things*, *Conditional and Perks.* Each of these motivations was found to have strong internal reliability. To the best of our knowledge, there is no known published research that has explicitly tested the underlying motivations of individuals who participate as ‘patient advisors’ in health care.

These findings are important for the future of patient engagement for three reasons. They suggest that individuals are motivated to not only satisfy needs, but also maximize the value they receive. Understanding motivations from the perspective of the patient or family member highlights what is important to them in their decisions to become engaged. This knowledge should lend itself to the design and delivery of productive and meaningful engagement programmes. Designing targeted engagement opportunities which provide value and meaning beyond ‘tokenistic’ involvement is key to the success of these initiatives. Second, research and other system‐wide initiatives involving patients and family members provide opportunities to further develop the skills and abilities of patients. Patients who are more ‘activated’ have the skills, ability and willingness to manage their own health and health care.^67^, ^68^, ^69^ This study served the dual purpose of promoting understanding of patient motivations and providing a concrete opportunity to enhance the capacity of patients to participate in health research. Third, these findings also highlight the importance of fair remuneration as a potential motivation for patients and family members who engage in this work. Purposely compensating individuals for their involvement reflects an ideological shift towards the patient as a true partner in health and health care.^70^, ^71^

Understanding the motivations of volunteers is not a novel area of research. Prior research on volunteer motivations has focused on understanding why people are motivated to help. This area of research continues to evolve and expand.^19^, ^21^, ^24^, ^25^, ^26^, ^72^, ^73^, ^74^, ^75^ Recognizing the motivations we identified are independent of each other and can be influenced by one or all seven identified.^36^ We found the *Self‐fulfillment* motivation primarily focuses on an individual's desire to find purpose,^25^, ^75^, ^76^, ^77^ to make connections^19^, ^21^, ^76^, ^78^, ^79^ and to help others^22^, ^32^, ^74^, ^79^ all while simultaneously benefiting from the experience.^75^, ^78^ The *Improving Healthcare* motivation highlights an individual's desire to ‘fix’ the health‐care system by improving not only the quality and service delivery^74^, ^80^, ^81^ but also perceived cultural challenges that are key to health‐care transformation, such as a lack of trust between patients and health‐care providers.^82^, ^83^, ^84^ The *Influence* motivation reflects an individual's ability to impact decisions, and to feel as though others are listening to them. The prestige of being associated with the health organization and associated feelings of pride (not only with themselves but with the work they are doing) further define this motivation^76^, ^77^ Having influence is key to the overall tenure of an individual's involvement in these initiatives. The *Learning New Things* motivation is fairly common in the volunteer literature and is primarily focused on an individual's desire to be exposed to new experiences and to have the chance to exercise knowledge, skills and abilities that might otherwise go unpracticed.^19^, ^25^, ^75^, ^76^, ^78^ The *Conditional* motivation describes how an individual makes a decision to participate, given a set of circumstances.^36^ For example, an individual who is unable to drive may find significant value in being able to participate in meetings held via teleconference. The decision to participate can be described as a balanced process where individuals weigh the potential benefits and risks of engaging in these endeavours.^85^

While our results generally support the findings in the literature, we did find some notable differences, specifically with respect to the *Compensation* and *Perks* motivations. Compensating individuals as research subjects occurs in some studies such as clinical trials.^86^, ^87^ It is also common for research studies to incent participation by offering a chance to win items such as gift cards. However, compensating patients as ‘partners’, as opposed to as research subjects, is an area that continues to expand and evolve.^70^, ^88^, ^89^, ^90^ Currently, in Canada, there are inconsistencies with how patient and family members are being compensated. The Canadian Institutes for Health Research (CIHR) under the Strategy for Patient‐Oriented Research is currently developing a guidance document to help researchers and others wanting to compensate individuals for their involvement.^91^ The *Perks* motivation is similar to compensation, but rather than being financially rewarded, individuals are provided extra benefits such as expense reimbursements or opportunities to attend conferences. This is a very interesting finding because it is contrary to the work of Deci^92^ which found negative effects on intrinsic motivation as a result of financial rewards. Our findings suggest that individuals engaged in these roles can be motivated by forms of compensation such as stipend payments, or extra benefits such as being supported to attend a conference. More research needs to be done to understand how these reimbursement techniques influence motivation and the importance of their role in patient engagement.

This study has limitations. First, we employed a cross‐sectional design which does not allow causal inferences to be made, or to assess changes over time. Second, a 0.50 cut‐off was used in factor identification to help maximize factor structure while maintaining exploratory conceptual fit. Given the varying recommendations provided for factor loading cut‐offs, future studies may elect to use a less stringent cut‐off.^50^, ^55^ It is important to note, however, that the findings from this current study may be the only known research on the motivations of patient and family volunteers. As such, our approach was designed to be a critical first step to rigorously identify patient motivations. Third, using only a five‐point Likert scale potentially reduced the overall reliability of these findings, and future research should attempt to confirm these results with a minimum 7‐point Likert scale.^93^ Fourth, only reliability was confirmed. Future research should include replicating this study via confirmatory factor analysis with a sample of participants with similar backgrounds. Fifth, this work focuses on the motivations of patient and family members. We acknowledge the importance of involving diverse groups of stakeholders in health‐care decision making, including clinicians and other health‐care professionals, and encourage future studies that explore and understand the motivations of these individuals. A final limitation of this study is that the results were based on a sample of patient and family members volunteering in various roles within one health‐care system and therefore do not necessarily allow the findings to be generalized across populations, or to other health‐care systems. Recognizing that the population is currently unknown and that this study represents the first of its kind, we attempted to ensure a reflective sample of individuals, from throughout the province, of patients and family members who engage with health systems in health‐care decision making. We found that individuals primarily participating in these roles are women, well‐educated, retired, married and living in urban centres. Volunteering trends support these findings, suggesting that some groups are more likely to volunteer than others.^94^, ^95^, ^96^ The results of the survey indicate the majority of individuals discovered the engagement opportunities by seeking it out themselves or were recommended by a friend of family member. This implies little to no recruitment efforts being undertaken by the respective health organizations. Broad participation of patient and family members is the most effective approach, providing legitimacy, creditability, transparency and accountability to any process. Equity should be the cornerstone of health care and often many of our health‐care challenges are driven by inequities in care. Given the universality of the MCB framework, we would postulate that the motivations among vulnerable and hard to reach populations would be ranked in a different level of priority and perhaps not all the motivations discovered would even be relevant within these groups. We know from Maslow's hierarchy of needs that when the lower level or foundational needs are not met, it is harder for individuals to think about higher level needs such as Influence or Self‐fulfillment.^14^, ^15^ Although our findings may reflect the population of usual advisors, there will be a time and place when seeking out the voices of hard to reach and vulnerable individuals will require deliberate strategies to support their inclusion and should be encouraged.^97^

Given the current interest in patient engagement coupled with the promising results of this study, more work needs to be done. Our results indicate that 33% of our respondents come from a health‐care background. This information could prove to be exceptionally valuable to health systems wanting to engage patients by understanding the kinds of individuals interested in engaging in this type of work and further research should explore this finding. As motivational research typically demonstrates variations within subgroups, future studies should attempt to determine whether patient and family members are motivated differently within groups. Research on patient participation in health‐care decision making would benefit from further explorations of the motivational commonalities and variations within rural and urban communities, ethnic groups, genders and socioeconomic classes Additional research needs to be completed on reimbursement strategies and their overall impact on patient participation in health care. Lastly, it is important to acknowledge our findings are based on a sample of patient and family members within one health‐care system and we must exercise caution in generalizing across populations, or other health‐care systems.

## 5 CONCLUSION

While significant research exists that highlights the motivations of people who volunteer, a limited number of studies have explored these concepts within health care. This study reports on the results of a provincial survey, describing a sample of patient partners currently occupying various roles within Alberta health organizations. We were able to identify seven motivations, which can be incorporated into a framework to explain and support future patient engagement initiatives. As the roles of patient and family advisors in the context of health‐care decision making continue to evolve, the importance of effective and sustainable engagement programmes will become increasingly important. The results of this study suggest that further research is needed to support the engagement of diverse groups of stakeholders, such as health‐care professionals and patient and family members, to assist in large‐scale health transformation. A deeper knowledge of patient motivations will not only create meaningful engagement opportunities for patients but will also enable health organizations to gain from the experience of these individuals. While further research is needed, the findings from this study have developed a preliminary understanding of the motivations of patients who engage in health‐care decision making.

## CONFLICT OF INTEREST

The authors declare that they have no competing interests.

## AUTHORS' CONTRIBUTIONS

TLM had significant involvement in the design, acquisition, analysis and interpretation of data. TN, NM, DH, DW and DL provided guidance in the overall design and delivery of the research. KM, SZ and GW were involved in the acquisition and final analysis. All authors provided revisions and the final approval to be published. All the named authors agree to take accountability for the integrity and accuracy of the work and have read and approved the final manuscript.

## ETHICAL APPROVAL

The ethics approval for the following research has been approved by the Conjoint Health Research Ethics Board at the University of Calgary.

## Data Availability

All data generated or analysed during this study are included in this published article.
