# Supplementary material for: Understanding the motivations of patients: A co‐designed project to understand the factors behind patient engagement
Source: Health Expect. 2019 Aug 4;22(4):709–20. doi: 10.1111/hex.12942 (PMC6737762; doi:10.1111/hex.12942)
Supplement: Supplementary file 1 [file HEX-22-709-s001.docx]

Supplementary Material: Participant survey

**SECTION 1: CONSENT**

**SECTION 2: YOUR ROLE**

In this section, we would like to find out more about your **primary role** with the healthcare system as a patient, family member or citizen.

- 1. Please select the role that best describes you:
- Volunteer
- Advisor
- Advocate
- Researcher
- Patient and Community Engagement Researcher (PaCER)
- Other: _____________________________
  1. How would you describe your current status?
- I am actively participating in this role
- I am currently taking a break
- I am no longer involved
- Other_____________
  1. How long have you been in this role (number of months)?

_______________________________

- 1. How do you feel about your role? For each statement**,** indicate your level of agreement, where 1=Strongly Disagree, 2=Disagree, 3=Neither Agree nor Disagree, 4=Agree, and
     5= Strongly Agree

|  | **Strongly Disagree** | **Disagree** | **Neither Agree nor Disagree** | **Agree** | **Strongly Agree** |
| --- | --- | --- | --- | --- | --- |
| 1. I feel proud | 1 | 2 | 3 | 4 | 5 |
| 1. I feel happy | 1 | 2 | 3 | 4 | 5 |
| 1. I feel stimulated | 1 | 2 | 3 | 4 | 5 |
| 1. I feel interested | 1 | 2 | 3 | 4 | 5 |
| 1. I feel underutilized | 5 | 4 | 3 | 2 | 1 |

2.5 How did you hear about this role? (Choose all that apply)?

- I received an email
- I was personally asked by my physician or healthcare provider
- I was contacted directly by an employee with (Alberta Health Services, Alberta Children’s Hospital, Covenant Health, etc.)
- My friend or family member told me about it
- I saw a poster or advertisement
- I searched out the opportunity myself
- Other: __________________________________
  1. Please list the different aspects of your role. We would like to understand what is it you actually do in your role? For example, I interview other patients about their experiences living with cancer; I participate in committee meetings as a patient representative on a Strategic Clinical Network; and I give comments and feedback on surveys to understand quality of care.
  2. On average, how much time do you spend in this role (hours per month)?
  3. If you were asked, would you be willing to give more time to this role?

Yes No

If no, why?

**SECTION 3: MAKING THE DECISION TO BE INVOLVED**

In this section, we would like to better understand how you made the decision to get involved as a patient, family member or citizen.

**3.1 How did you learn about what was required of you in this role? (Choose all that apply)**

- I participated in training
- I went through a selection process (an interview and/or an application form)
- I attended an information/orientation session
- I researched the opportunity online
- My friend told me about it
- I was asked by my physician to consider the opportunity
- Other: _________________
  1. **Why did you decide to get involved?** For each statement**,** indicate your level of agreement, where 1=Strongly Disagree, 2=Disagree, 3=Neither Agree nor Disagree, 4=Agree, and
     5= Strongly Agree

|  | **Strongly Disagree** | **Disagree** | **Neither Agree nor Disagree** | **Agree** | **Strongly Agree** |
| --- | --- | --- | --- | --- | --- |
| 1. It is an opportunity to make some extra money | 1 | 2 | 3 | 4 | 5 |
| 1. I get to travel | 1 | 2 | 3 | 4 | 5 |
| 1. I want to improve healthcare for myself and my family | 1 | 2 | 3 | 4 | 5 |
| 1. I feel I can make a contribution | 1 | 2 | 3 | 4 | 5 |
| 1. I get to learn new things | 1 | 2 | 3 | 4 | 5 |
| 1. I want to improve the health care system | 1 | 2 | 3 | 4 | 5 |
| 1. Other, please specify |  |  |  |  |  |

- 1. **How influential were the following on your decision to be involved?** For each statement**,** indicate your level of agreement, where 1=Strongly Disagree, 2=Disagree, 3=Neither Agree nor Disagree, 4=Agree, and 5= Strongly Agree

|  | **Strongly Disagree** | **Disagree** | **Neither Agree nor Disagree** | **Agree** | **Strongly Agree** |
| --- | --- | --- | --- | --- | --- |
| 1. Being part of a team | 1 | 2 | 3 | 4 | 5 |
| 1. Earning extra money | 1 | 2 | 3 | 4 | 5 |
| 1. Improving the healthcare system | 1 | 2 | 3 | 4 | 5 |
| 1. Learning new things | 1 | 2 | 3 | 4 | 5 |
| 1. Working with influential health professionals | 1 | 2 | 3 | 4 | 5 |
| 1. Convenience – I can do what is needed from the comfort of my own home | 1 | 2 | 3 | 4 | 5 |
| 1. Other, please specify |  |  |  |  |  |

- 1. **Prior to starting this role, what difference did you hope to make?** For each statement**,** indicate your level of agreement, where 1=Strongly Disagree, 2=Disagree, 3=Neither Agree nor Disagree, 4=Agree, and 5= Strongly Agree

|  | **Strongly Disagree** | **Disagree** | **Neither Agree nor Disagree** | **Agree** | **Strongly Agree** |
| --- | --- | --- | --- | --- | --- |
| 1. To make healthcare better | 1 | 2 | 3 | 4 | 5 |
| 1. To change the current culture of healthcare | 1 | 2 | 3 | 4 | 5 |
| 1. To be recognized as a partner | 1 | 2 | 3 | 4 | 5 |
| 1. To speak for those who can’t speak for themselves | 1 | 2 | 3 | 4 | 5 |
| 1. To impact decisions how decisions are made | 1 | 2 | 3 | 4 | 5 |
| 1. Other, please specify |  |  |  |  |  |

**SECTION 4: YOUR EXPERIENCE**

In this section, we would like to better understand your experience while serving in your current role as a patient, family member or citizen.

- 1. **In this role, I feel ____________.** For each statement**,** indicate your level of agreement, where 1=Strongly Disagree, 2=Disagree, 3=Neither Agree nor Disagree, 4=Agree, and 5= Strongly Agree

|  | **Strongly Disagree** | **Disagree** | **Neither Agree nor Disagree** | **Agree** | **Strongly Agree** |
| --- | --- | --- | --- | --- | --- |
| 1. Needed | 1 | 2 | 3 | 4 | 5 |
| 1. I have wasted my time | 5 | 4 | 3 | 2 | 1 |
| 1. Appreciated | 1 | 2 | 3 | 4 | 5 |
| 1. Excited | 1 | 2 | 3 | 4 | 5 |
| 1. Challenged | 1 | 2 | 3 | 4 | 5 |
| 1. Important | 1 | 2 | 3 | 4 | 5 |
| 1. I have made a difference | 1 | 2 | 3 | 4 | 5 |
| 1. Valued | 1 | 2 | 3 | 4 | 5 |
| 1. Unappreciated | 5 | 4 | 3 | 2 | 1 |
| 1. Included | 1 | 2 | 3 | 4 | 5 |
| 1. Engaged | 1 | 2 | 3 | 4 | 5 |
| 1. Other, please specify |  |  |  |  |  |

- 1. **Doing this work gives me a sense of ­­­­­­­­­­­_______________**. For each statement**,** indicate your level of agreement, where 1=Strongly Disagree, 2=Disagree, 3=Neither Agree nor Disagree, 4=Agree, and 5= Strongly Agree

|  | | **Strongly Disagree** | **Disagree** | | **Neither Agree nor Disagree** | **Agree** | **Strongly Agree** |
| --- | --- | --- | --- | --- | --- | --- | --- |
| 1. Purpose | 1 | | | 2 | 3 | 4 | 5 |
| 1. Aggravation | 5 | | | 4 | 3 | 2 | 1 |
| 1. Competence | 1 | | | 2 | 3 | 4 | 5 |
| 1. Knowledge | 1 | | | 2 | 3 | 4 | 5 |
| 1. Empowerment | 1 | | | 2 | 3 | 4 | 5 |
| 1. Inspiration | 1 | | | 2 | 3 | 4 | 5 |
| 1. Frustration | 5 | | | 4 | 3 | 2 | 1 |
| 1. Connection | 1 | | | 2 | 3 | 4 | 5 |
| 1. Hope | 1 | | | 2 | 3 | 4 | 5 |
| 1. Other, please specify |  | | |  |  |  |  |

- 1. **What benefits have you received from this opportunity?** For each statement**,** indicate your level of agreement, where 1=Strongly Disagree, 2=Disagree, 3=Neither Agree nor Disagree, 4=Agree, and 5= Strongly Agree

|  | **Strongly Disagree** | **Disagree** | **Neither Agree nor Disagree** | **Agree** | **Strongly Agree** |
| --- | --- | --- | --- | --- | --- |
| 1. A sense of purpose | 1 | 2 | 3 | 4 | 5 |
| 1. Helping others | 1 | 2 | 3 | 4 | 5 |
| 1. Meeting influential people | 1 | 2 | 3 | 4 | 5 |
| 1. Helping to improve healthcare | 1 | 2 | 3 | 4 | 5 |
| 1. I am paid | 1 | 2 | 3 | 4 | 5 |
| 1. I learn new things | 1 | 2 | 3 | 4 | 5 |
| 1. I am more informed about healthcare | 1 | 2 | 3 | 4 | 5 |
| 1. I get to travel | 1 | 2 | 3 | 4 | 5 |
| 1. My expenses are paid | 1 | 2 | 3 | 4 | 5 |
| 1. Other, please specify: |  |  |  |  |  |

**SECTION 5: YOUR CONTRIBUTION**

In this section, we would like to better understand the contribution you have had in your role as a patient, family member or citizen.

- 1. **How influential do you think you are in your role**? For each statement**,** indicate your level of agreement, where 1=Strongly Disagree, 2=Disagree, 3=Neither Agree nor Disagree, 4=Agree, and 5= Strongly Agree

|  | **Strongly Disagree** | **Disagree** | | **Neither Agree nor Disagree** | **Agree** | | **Strongly Agree** | |
| --- | --- | --- | --- | --- | --- | --- | --- | --- |
| 1. Others listen to me | 1 | 2 | 3 | | | 4 | | 5 |
| 1. I do not feel influential | 5 | 4 | 3 | | | 2 | | 1 |
| 1. I am impacting decisions | 1 | 2 | 3 | | | 4 | | 5 |
| 1. I am challenging the “norm” | 1 | 2 | 3 | | | 4 | | 5 |
| 1. I am paving the way for others | 1 | 2 | 3 | | | 4 | | 5 |
| 1. I feel I am making a difference | 1 | 2 | 3 | | | | 4 | 5 |
| 1. Other, please specify |  |  |  | | | |  |  |

- 1. **Thinking about your contributions so far, what impact do you think you have had?** For each statement**,** indicate your level of agreement, where 1=Strongly Disagree, 2=Disagree, 3=Neither Agree nor Disagree, 4=Agree, and 5= Strongly Agree

|  | **Strongly Disagree** | **Disagree** | **Neither Agree nor Disagree** | **Agree** | **Strongly Agree** |
| --- | --- | --- | --- | --- | --- |
| 1. Communication between patients/family members and health professionals have improved | 1 | 2 | 3 | 4 | 5 |
| 1. I have not had much of an impact | 5 | 4 | 3 | 2 | 1 |
| 1. I have improved patient experience | 1 | 2 | 3 | 4 | 5 |
| 1. I have established important relationships | 1 | 2 | 3 | 4 | 5 |
| 1. I have seen the beginning of a shift in healthcare | 1 | 2 | 3 | 4 | 5 |
| 1. Other, please specify |  |  |  |  |  |

**Section 6: ONGOING INVOLVEMENT**

In this section, we would like to better understand the longevity of your role as a patient, family member or citizen.

- 1. **Why do you continue to do this role?** For each statement**,** indicate your level of agreement, where 1=Strongly Disagree, 2=Disagree, 3=Neither Agree nor Disagree, 4=Agree, and 5= Strongly Agree

|  | **Strongly Disagree** | **Disagree** | **Neither Agree nor Disagree** | **Agree** | **Strongly Agree** |
| --- | --- | --- | --- | --- | --- |
| 1. I am making a difference | 1 | 2 | 3 | 4 | 5 |
| 1. I meet new people | 1 | 2 | 3 | 4 | 5 |
| 1. I am supporting other patients | 1 | 2 | 3 | 4 | 5 |
| 1. I get to work on new things | 1 | 2 | 3 | 4 | 5 |
| 1. I am getting paid | 1 | 2 | 3 | 4 | 5 |
| 1. This work could lead to future employment | 1 | 2 | 3 | 4 | 5 |
| 1. Other, please specify |  |  |  |  |  |

- 1. **How important are the following aspects of this role in supporting your continued involvement**? For each statement**,** indicate your level of agreement, where 1=Strongly Disagree, 2=Disagree, 3=Neither Agree nor Disagree, 4=Agree, and 5= Strongly Agree

|  | **Strongly Disagree** | **Disagree** | **Neither Agree nor Disagree** | **Agree** | **Strongly Agree** |
| --- | --- | --- | --- | --- | --- |
| 1. Senior healthcare leaders support this work | 1 | 2 | 3 | 4 | 5 |
| 1. I see the difference I am making | 1 | 2 | 3 | 4 | 5 |
| 1. The projects are interesting | 1 | 2 | 3 | 4 | 5 |
| 1. I receive recognition | 1 | 2 | 3 | 4 | 5 |
| 1. I receive payment | 1 | 2 | 3 | 4 | 5 |
| 1. I am able to travel | 1 | 2 | 3 | 4 | 5 |
| 1. The role is flexible | 1 | 2 | 3 | 4 | 5 |
| 1. I can work from home | 1 | 2 | 3 | 4 | 5 |
| 1. I enjoy what I am doing | 1 | 2 | 3 | 4 | 5 |
| 1. I continue to learn | 1 | 2 | 3 | 4 | 5 |
| 1. My expenses are paid | 1 | 2 | 3 | 4 | 5 |
| 1. I get to meet new people | 1 | 2 | 3 | 4 | 5 |
| 1. Other, please specify |  |  |  |  |  |

- 1. **Would you like this role to continue?**
- Yes
- No

- 1. **If yes, how much longer would you like to be involved in this role?**

**If no, please tell us why?**

- 1. **Let’s imagine a new opportunity presented itself in a different area within healthcare**. **To what extent would the following conditions impact your decision to accept this new opportunity?** For each statement**,** indicate your level of agreement, where 1=Strongly Disagree, 2=Disagree, 3=Neither Agree nor Disagree, 4=Agree, and 5= Strongly Agree

|  | **Strongly Disagree** | **Disagree** | **Neither Agree nor Disagree** | **Agree** | **Strongly Agree** |
| --- | --- | --- | --- | --- | --- |
| 1. The commitment requires that you only attend four meetings a year | 1 | 2 | 3 | 4 | 5 |
| 1. You are the only patient or family representative | 1 | 2 | 3 | 4 | 5 |
| 1. Your expenses are reimbursed | 1 | 2 | 3 | 4 | 5 |
| 1. You can work from home | 1 | 2 | 3 | 4 | 5 |
| 1. You do not need to travel | 1 | 2 | 3 | 4 | 5 |
| 1. The role could turn into a paid position | 1 | 2 | 3 | 4 | 5 |
| 1. You attend an annual conference | 1 | 2 | 3 | 4 | 5 |
| 1. Other |  |  |  |  |  |

**SECTION 7: DEMOGRAPHIC INFORMATION**

This section of the survey is to make sure we have a balanced representation throughout the province. Remember your responses are anonymous. No individual responses will be shared. All responses will be aggregated and analyzed.

- 1. I consider myself:
- Male
- Female
- I prefer not to answer
- Other, please specify
  1. How old are you?
- ________
- I prefer not to answer
  1. What is the highest level of education you have completed? If currently enrolled, highest degree received:
  - Secondary School
  - High School
  - College
  - University – Bachelor
  - University – Graduate (Masters, PhD)
  - None
  - Other, please specify
  - I prefer not to answer
  1. I am:
  - Single
  - Married (and not separated)
  - Common-law
  - Separated, but still legally married
  - Divorced
  - Widowed
  - I prefer not to answer
  - Other, please specify
  1. Where do you live? (drop down menu)
  - Airdrie
  - Brooks
  - Calgary
  - Camrose
  - Canmore
  - Chestermere
  - Cochrane
  - Cold Lake
  - Edmonton
  - Grande Prairie
  - High River
  - Fort McMurray/Wood Buffalo
  - Fort Saskatchewan
  - Lacombe
  - Leduc
  - Lethbridge
  - Lloydminster
  - Medicine Hat
  - Red Deer
  - Spruce Grove
  - St. Albert
  - Sherwood Park
  - I prefer not to answer
  - Other, please specify
  1. Which of these describes you (CHOOSE ALL THAT APPLY)?
  - Full-time employed
  - Part-time employed
  - Caregiver
  - Homemaker
  - Full-time student
  - Part-time student
  - Self-employed
  - Receiving disability benefits
  - Retired
  - I prefer not to answer
  - Other, please specify
  1. Have you previously worked in the healthcare system?
- Yes
- No
  1. If yes, what was your role?

**SECTION 8: ADDITIONAL OPPORTUNITIES**

**Stay connected!**

If you would like to be contacted about additional opportunities to participate in this project please enter your email address.

Please note, this information will not be attached to your survey and your results will remain completely anonymous.

Please enter your email address:

| Email: |  |
| --- | --- |

**Enter for a chance to win!**

Thank you for participating in our survey. If you would like to be entered into a draw for a chance to win one of 2 - $100 VISA gift cards please enter your email address below.

A random draw of completed surveys will be conducted within a week of the closing date. Please note only the winners will be contacted.

Please enter your email address:

| Email: |  |
| --- | --- |
